# Supplementary material for: Beyond the Numbers: Quantitative and qualitative analysis of quality of life after deep brain stimulation for OCD
Source: PLoS One. 2026 May 29;21(5):e0350293. doi: 10.1371/journal.pone.0350293 (PMC13221007; doi:10.1371/journal.pone.0350293)
Supplement: Attachment — Submitted filename: Response to Reviewers.docx [file pone.0350293.s003.docx]

Response to editor and reviewers:

Thank you for your feedback and careful review of our manuscript. We have completed all revisions and our responses to each can be found below.

Editor

Please include the following items when submitting your revised manuscript:

- A letter that responds to each point raised by the academic editor and reviewer(s). You should upload this letter as a separate file labeled 'Response to Reviewers'. **Completed**
- A marked-up copy of your manuscript that highlights changes made to the original version. You should upload this as a separate file labeled 'Revised Manuscript with Track Changes'. **Completed**
- An unmarked version of your revised paper without tracked changes. You should upload this as a separate file labeled 'Manuscript'. **Completed**

Journal Requirements:

When submitting your revision, we need you to address these additional requirements.

1. Please ensure that your manuscript meets PLOS ONE's style requirements, including those for file naming. **Completed**

2. Thank you for stating the following in the Competing Interests section: “I have read the journal's policy and the authors of this manuscript have the following competing interests: Rachel Davis provides paid consultation for Medtronic.” ***This does not alter our adherence to PLOS ONE policies on sharing data and materials.*** **Completed**

3. We note that your Data Availability Statement is currently as follows: “All relevant data are within the manuscript and its Supporting Information files.” ***Additional de-identified data has been added to the supporting information files. See supporting information file for a summary sheet with days post stimulation, Y-BOCS and QoL percent change values***. **Completed**

4. If the reviewer comments include a recommendation to cite specific previously published works, please review and evaluate these publications to determine whether they are relevant and should be cited. There is no requirement to cite these works unless the editor has indicated otherwise.  **Completed**

**Reviewer #1:**

1. There are two recent (2025) meta-analyses on DBS for OCD that may provide a more complete and actual overview of the literature, which could be added to the introduction.

***We agree that these references add a more robust and more current overview of the literature. These references and related information were added to the introduction and are listed under reference numbers [31] and [32]. Lines 83-89 (tracked changes manuscript).***

2. The authors do not cite the largest cohort of DBS for OCD published to date, which may be relevant to their introduction (Denys et al., Am J Psych, 2020, with a follow-up also on QoL in Graat et al., Biol Psych, 2021).

***We agree with the reviewer about the inclusion of these references, especially Graat et al., 2021. These references and related information were added to the introduction and are listed under reference numbers [30] and [33]. Lines 83-89 (tracked changes manuscript).***

3. Line 185-187: The authors describe the calculation of a baseline Y-BOCS, but do not precisely describe how this calculation was performed. Did they take the mean of the 1 to 3 pre-DBS Y-BOCS values or did they perform a different calculation?

***We calculated the average of the pre-DBS Y-BOCS baseline days. If only 1 day was collected during the pre-DBS period, then that day was used as the baseline.***

4. Line 189-190: the authors describe the number of follow-up sessions in days, which seems incorrect.

***This was edited for clarity and now appears in lines 216-217 (tracked changes manuscript)/210-211 clean copy: “As subjects varied in the number of follow-up sessions over the course of 974-3203 days after stimulation was turned on, non-parametric summary…”***

5. The authors describe and show that they divided the number of follow-up sessions in quartiles per patient (189-190 and figure 2). This may be somewhat misleading as the number of follow-up sessions differs between patients (973-3203). For example Q4 may include follow-up up to day 973 in one patient and up to 3203 in another. These large differences in follow-up in one X-axis label may distort interpretations. The authors may try to use a different X-axis labelling, e.g. months since start of stimulation.

***We have updated Figure 2 to include the size of each subject’s quarter percent change marker to account for number days during quarter. See new Figure 2.***

6. Line 191-192, Figure 3: the authors use Pearson correlations to test the within-subject correlation between percent change in Y-BOCS and Q-LES-Q-SF. Given that these are repeated measures within subject over time, there likely is autocorrelation, which may influence the correlation. In addition, the authors now only tested the within-subject correlation. The authors may consider using linear mixed models to test the association taking autocorrelation into account and test significance across subjects.

***We agree with the reviewer’s concern and have replaced the current analysis using Pearson correlations with a linear mixed model using Q-LES-Q-SF as a fixed effect, subject as a random intercept and included a continuous autoregressive correlation to account for time between measurements. The LMM confirmed a significant association between symptom reduction and quality of life improvement (β= -0.15, p < .001), demonstrating that the relationship remains robust after accounting for the repeated measures structure and autocorrelation. We have updated the Methods and Results sections (Lines 219-223 tracked changes manuscript/212-216 clean copy) to reflect this more rigorous analysis.***

7. Line 196-208: do the authors have references supporting the methodology to analyze the qualitative data?

***We have updated the methodology procedure section to include references supporting the methodologies used, inductive analysis and thematic analysis, to analyze the qualitative data (232-235 tracked changes manuscript/224-228 clean copy).***

8. The authors report on observational quantitative and qualitative data, did the authors consider the use of reporting guidelines such as STROBE and/or SRQR?

***The reviewer correctly states that our data is both quantitative and qualitative. A formal checklist was not submitted with our research as it was not mandatory. Due to the mixed methods nature of this study, the general guidelines for SRQR and STROBE were both followed when conceptualizing the manuscript. The team can provide an official checklist if reviewers request this to be submitted.***

9. The authors report quotes in the discussion, which may be better seen as results.

***The authors agree with this reviewer’s recommendation and have moved most of the quotes into the results section, including Table 4.***

10. The authors report several recommendations for clinical care based on their results. Given the small N and the observational nature of the data, the authors should tone down their tone and present their findings in the context of broader data. For example, while the importance of post-DBS ERP is intuitive and reported in the qualitative data, more controlled data did not unambiguously show additional effects (e.g. Graat et al., Psych Med, 2022).

***We agree with this reviewer’s statement as our population was small in this research. We went through out results and discussion and worked on the language to change the tone of our clinical recommendations per the reviewer’s comments. We are unsure if the reviewer was referencing the previously mentioned Graat et al., 2021 or a different study, as we were unable to locate the listed Graat et al., Psych Med, 2022.***

11. Line 366-367: in their conclusion, the authors note “It should not be assumed, based on quantitative measures alone, that QoL has not improved or has only minimally improved in participants with OCD post-DBS.” However, the authors do not present data on the general (e.g. mean) overall improvement of QoL in their sample. The authors may consider adding this data, also including e.g. percentage of (partial) responders.

***The percentage change from baseline in QoL was added to the results section to address this point. The team also attached the supporting information file that includes a summary sheet with days post stimulation, Y-BOCS and QoL percent change values. Lines 255-258 (tracked changes manuscript) or 245-248 (clean copy).***

**Reviewer #2:** 

**Abstract:**
Authors should clarify that analyses were completed within each participant, using solely their data points, in order to assess for individual correlations

- ***This was addressed in the “methods” section of the abstract.***

**Introduction:**
The introduction, while it covers relevant information, is a bit choppy, with some very short (i.e. 2 sentence) paragraphs

- ***The introduction was reviewed and paragraphs were lengthened to not be as short.***

The final hypothesis should be clarified- “Patients will report similar improvements in QoL themes.” Were there particular themes that the author anticipated would improve versus others?

- ***More details were added to this hypothesis that indicate the authors anticipated themes of improvement in OCD and QoL (Lines 129-130 tracked changes manuscript/123-125 clean copy).***

**Methods:**
It should not be necessary to include specific numerical dates for initial assessments, date of ethics determination, closure date, and date of acquisition of quantitative data, particularly given the small sample size. Either year or month and year (without date) should be sufficient.

- ***Per this reviewer’s suggestions, the dates for the above were simplified to month and year.***

Authors should not include the coding system for their deidentification, as this is providing information to a global audience that theoretically could lead to patient identification.

- ***The specific coding system for deidentification was removed.***

On page 6, “quantitative assessments and reviews of this data” should be “quantitative assessments and review of these data.”

- ***This has been corrected.***

The method and related IRB approvals is unclear. If this is a retrospective analysis of already-collected clinical data, only a PHI waiver from the IRB should be necessary. However, it seems as if the qualitative analysis was added on and NOT part of the clinical procedures, just research. Thus, patients should be consented for this. The writing is not clear- it first states that no ethical approval was needed but then states that participants provided informed consent. Please clarify.

- ***The writers edited this piece of the “Methods” section to clarify that the study was deemed exempt. The quantitative data was part of clinical procedures and did not require informed consent per the IRB’s decision. However, the IRB required patients to provide informed consent for the additional qualitative interview data collection, as well as the use of the quantitative data for research and publication. Lines 166-172 (tracked changes manuscript) or 161-167 (clean copy).***

Given that individual correlations were conducted, how many data points (range across patients) were in the correlations?

- ***In response to a comment by R1 (#6) we have updated the analysis approach to the relationship between mood and OCD symptoms. We have replaced individual Pearson correlations with a linear mixed model that also attempts to address a confound related to the temporal relationship of the data. Briefly, using this approach we confirmed a significant association between symptom reduction and quality of life improvement (β= -0.15, p < .001), demonstrating that the relationship remains robust after accounting for the repeated measures structure and autocorrelation. We have updated the Methods and Results sections to reflect this more rigorous analysis. Lines 219-223 tracked changes manuscript/212-216 clean copy.***

**Results:**
Given that there is an extended period of follow-up per patient after DBS, authors should describe time line of improvements in YBOCS score and QoL For example, on page 9, authors note “Eight of ten participants reported that life improved following the DBS procedure.” What is the time frame on this? Did it persist? Were improvements in QoL and DBS based on last follow-up, any time point, etc?

- ***At the time of the interview, eight of ten participants reported that life improved following the DBS procedure. The timeframe between initial DBS procedure and this interview varied between individual participants However, we added additional information from the qualitative data that speaks to when participants noticed the changes and whether or the changes were long lasting for them. Table 3 was also added to provide a summary of participant responses about timeframe and longevity of impact. The team also attached the supporting information file that includes a summary sheet with days post stimulation, Y-BOCS and QoL percent change values.***

Authors randomly put in that there were no changes in vision or dizziness following DBS- was that part of the QoL measure, or a statement about side effects in general?

- ***Vision and dizziness are measured on the Q-LES-Q-SF, which was the measure used to assess QoL. However, for clarity and conciseness, authors removed this from the results section as it did not add to the writing.***

Were the timelines of changes in YBOCS and QoL different after surgery? We may anticipate that recognition of QoL may be somewhat delayed as compared to symptom improvement, which may impact correlations. Was this examined?

- ***At the time of the interview, eight of ten participants reported that life improved following the DBS procedure. The timeframe between initial DBS procedure and this interview varied between individual participants. The team attached the supporting information file that includes a summary sheet with days post stimulation, Y-BOCS and QoL percent change values. Figure 4 was added to capture the rate of change. The rate of change in either metric is quite variable and is captured in the quartile plot (and individual subject change over time plots). However, Figure 4 shows a higher resolution plot (30 day medians)(B). We also included a representative subject (subject 7) that the QoL is more variable than YBOCS (A). Table 3 was also added to provide qualitative data regarding timelines of change as well as long-lasting impact of DBS.***

**Discussion:**
Authors note “Patients may retrospectively recognize that their impaired mental state…post-DBS.” Are they indicating that there are ethical concerns about these patients receiving DBS? This is what this sounds like in reading this sentence.

- ***The cited studies [41, 52-56] after this sentence discuss a patient’s impaired mental state pre-surgery as a potential ethical concern in regard to their ability to fully understand the outcome of DBS. Previous research has not shown substantial data that this would be an ethical concern regarding consent. The words “impaired”, “risks” and “benefits” were removed to change the tone of the sentence. Lines 409-411 (tracked changes manuscript) or 386-387 (clean copy).***

1. ***Acevedo N, Castle DJ, Bosanac P, Groves C, Rossell SL. Patient feedback and psychosocial outcomes of deep brain stimulation in people with obsessive–compulsive disorder. J Clin Neurosci. 2023;112:80–5. doi:10.1016/j.jocn.2023.04.012.***
2. ***Maier, F., Lewis, C. J., Horstkoetter, N., Fink, G. R., Timmermann, L., & Faust, P. (2013). Patients’ expectations of deep brain stimulation, and subjective perceived outcome related to clinical measures in Parkinson's disease: A mixed-method approach. Journal of Neurology, Neurosurgery & Psychiatry, 84(11), 1273–1281.*** [***https://doi.org/10.1136/jnnp-2012-303670***](https://doi.org/10.1136/jnnp-2012-303670)
3. ***Hasegawa, H., Samuel, M., Douiri, A., & Ashkan, K. (2014). Patients' expectations in subthalamic nucleus deep brain stimulation surgery for Parkinson disease. World Neurosurgery, 82(6), 1295–1299.e2.*** [***https://doi.org/10.1016/j.wneu.2014.02.001***](https://doi.org/10.1016/j.wneu.2014.02.001)
4. ***Thomson, C., & Carter, A. (2020). Ethical issues in experimental treatments for psychiatric disorders: Lessons from deep brain stimulation. Translational Issues in Psychological Science, 6(3), 240–246.*** [***https://doi.org/10.1037/tps0000267***](https://doi.org/10.1037/tps0000267)
5. ***Acevedo, N., Castle, D., Groves, C., Bosanac, P., Mosley, P. E., & Rossell, S. (2022). Clinical recommendations for the care of people with treatment-refractory obsessive-compulsive disorder when undergoing deep brain stimulation. Australian & New Zealand Journal of Psychiatry, 56(10), 1219–1225.*** [***https://doi.org/10.1177/00048674221100947***](https://doi.org/10.1177/00048674221100947)
6. ***Martinho, F. P., Duarte, G. S., & do Couto, F. S. (2020). Efficacy, effect on mood symptoms, and safety of deep brain stimulation in refractory obsessive-compulsive disorder: A systematic review and meta-analysis. The Journal of Clinical Psychiatry, 81(3), 4067.*** [***https://doi.org/10.4088/JCP.19r12821***](https://doi.org/10.4088/JCP.19r12821)

Quotes should likely be in the Results section if they are part of a qualitative analysis; there are an extensive number of quotes in the discussion that may be better placed elsewhere.
It appears that there was a push from the investigators to have the patients assess their QoL in a different way; authors should be very careful about this, as this may be interpreted as the clinical providers placing undue pressure on the patients in order to say that their QoL was actually better than initially reported (e.g. page 13 “Only after building insight and being asked to zoom out to see the bigger picture…overall symptoms and lives.”)

- ***The authors agree with the feedback to move the location of the quotes. Most of the quotes were moved to the results section. The quote listed by reviewer was revised to better represent what authors were trying to capture with that sentence.***

**Figures:**
Figure 1 is unclear. It looks like all except subject 1 started with a change in YBOCS score to begin with? It also appears that most participants had an initial drop in YBOCS and then an increase later- how many patients were long-term responders? Similar questions for QoL.

- ***We thank the reviewer for this question. However, it appears that the visualization has allowed the reviewer to draw the correct inference and main message. That is, most subjects show a response, however, there is fluctuation in the magnitude of the response over time. This visualization is not intended to convey long term vs short responders, since all subjects show a change from baseline that persists over time. We have added the relevant responder rate information to the text and figures in the results section.***

There is a similar question for Figure 2- for the first data point, was this after xx days of stimulation? Sounds like after a quartile of stim- which I assume could be very different depending on follow-up period?

- ***We have updated this plot to convey the number of days of stimulation in each quarter using the size of the scatter point. There is a legend in the plot to indicate how to interpret days.***

Figure 3 is unclear. Is the y-axis change in QoL, and the x-axis YBOCS change? They don’t look like raw scores, but it is unclear. Also, if possible, scales across patients should be consistent.

- ***We have replaced this Figure with a new summary Figure and have clarified that the x and y axes are percent change from baseline.***
