# Supplementary material for: Beyond the Numbers: Quantitative and qualitative analysis of quality of life after deep brain stimulation for OCD
Source: PLoS One. 2026 May 29;21(5):e0350293. doi: 10.1371/journal.pone.0350293 (PMC13221007; doi:10.1371/journal.pone.0350293)
Supplement: Attachment — Submitted filename: Response to Reviewers 4.26.docx [file pone.0350293.s004.docx]

Response to editor and reviewers:

Thank you for your feedback and careful review of our manuscript. We have completed all revisions and our responses to each can be found below. These are the updated revisions that were provided to our team after our February 2026 submission.

Editor

Please include the following items when submitting your revised manuscript:

- A letter that responds to each point raised by the academic editor and reviewer(s). You should upload this letter as a separate file labeled 'Response to Reviewers'. **Completed**
- A marked-up copy of your manuscript that highlights changes made to the original version. You should upload this as a separate file labeled 'Revised Manuscript with Track Changes'. **Completed**
- An unmarked version of your revised paper without tracked changes. You should upload this as a separate file labeled 'Manuscript'. **Completed**

Journal Requirements:

1. If the reviewer comments include a recommendation to cite specific previously published works, please review and evaluate these publications to determine whether they are relevant and should be cited. There is no requirement to cite these works unless the editor has indicated otherwise.  **Completed.**

2. Please review your reference list to ensure that it is complete and correct. If you have cited papers that have been retracted, please include the rationale for doing so in the manuscript text, or remove these references and replace them with relevant current references. Any changes to the reference list should be mentioned in the rebuttal letter that accompanies your revised manuscript. If you need to cite a retracted article, indicate the article’s retracted status in the References list and also include a citation and full reference for the retraction notice. **Completed. All references have been checked and as of 4/8/2026, there are no retracted or incorrect references that the team found upon multiple reviews and checks. References that were in the incorrect citation format were corrected.**

**Reviewer #1:**


3. Could the authors include the explanation on the calculation of the baseline Y-BOCS in the manuscript?

***Baseline Y-BOCS was defined as the mean of all available pre-DBS assessments to provide a representative estimate of pre-surgical symptom severity. In treatment-refractory OCD, symptom severity can fluctuate over time due to intrinsic variability and ongoing treatment adjustments, and a single pre-operative timepoint may reflect a transient clinical state rather than typical illness severity. Averaging across multiple pre-operative measurements reduces the influence of short-term variability. This clarification has been added to the manuscript in the methods section.***

5. I still think figure 2 is hard to interpret, because strongly different durations of follow-up are compared. In addition, I don’t understand how the number of days in the quartiles can differ within a subject. I strongly suggest to make more thorough changes in this figure, e.g. in line with the previous suggestion.

***Figure 2 was revised further. We thank the reviewer for this additional feedback regarding figure clarity. We have revised Figure 2 in the following aspects. 1) We removed the quartiles and represented each subject with their metric at each assessment as a function of months post stimulation onset. 2) We removed the change in increase or decrease in the metric based on color change. 3) We have added linear least square regression lines for each metric across subjects (up to 72 months).***

10. The reference can be found here: Cognitive behavioral therapy in patients with deep brain stimulation for obsessive-compulsive disorder: a matched controlled study | Psychological Medicine | Cambridge Core

***We thank the reviewer for providing the reference information. This reference has been added to the discussion section (line 366 in clean manuscript, line 385 in tracked changes manuscript)***
