# Supplementary material for: Beyond the Numbers: Quantitative and qualitative analysis of quality of life after deep brain stimulation for OCD
Source: PLoS One. 2026 May 29;21(5):e0350293. doi: 10.1371/journal.pone.0350293 (PMC13221007; doi:10.1371/journal.pone.0350293)
Supplement: Attachment — Submitted filename: Response to Reviewers 5.7.docx [file pone.0350293.s005.docx]

Response to editor and reviewers:

Thank you for your feedback and careful review of our manuscript. We have completed all revisions and our responses to each can be found below. These are the updated revisions that were provided to our team after our February 2026 submission.

Editor

Please include the following items when submitting your revised manuscript:

- A letter that responds to each point raised by the academic editor and reviewer(s). You should upload this letter as a separate file labeled 'Response to Reviewers'. **Completed**
- A marked-up copy of your manuscript that highlights changes made to the original version. You should upload this as a separate file labeled 'Revised Manuscript with Track Changes'. **Completed**
- An unmarked version of your revised paper without tracked changes. You should upload this as a separate file labeled 'Manuscript'. **Completed**

Journal Requirements:

1. If the reviewer comments include a recommendation to cite specific previously published works, please review and evaluate these publications to determine whether they are relevant and should be cited. There is no requirement to cite these works unless the editor has indicated otherwise.  **Completed.**

2. Please review your reference list to ensure that it is complete and correct. If you have cited papers that have been retracted, please include the rationale for doing so in the manuscript text, or remove these references and replace them with relevant current references. Any changes to the reference list should be mentioned in the rebuttal letter that accompanies your revised manuscript. If you need to cite a retracted article, indicate the article’s retracted status in the References list and also include a citation and full reference for the retraction notice. **Completed. All references have been checked and as of 4/8/2026, there are no retracted or incorrect references that the team found upon multiple reviews and checks. References that were in the incorrect citation format were corrected.**

**Reviewer #1:**


Reviewer #1: The authors have further improved the manuscript. One final point remains which seems not fully covered by the revisions made: the figures.

1. In the revised manuscript, both figure 1 and 2 show the trajectories of individual Y-BOCS and QLES scores over time. But the trajectories of the lines seem quite different between the figures, how could that be explained, given that they are from the same individuals?

***The Figures represent the same information in slightly different formats. We appreciate that the inclusion of both figures may be confusing, so we have decided to Remove Figure 1.***

2. Moreover, the revised figure 4 is less confusing, but it remains unclear in the figure that the number of participants during follow-up decreases (which influences the IQR). This should be noted in the figure legend.

***We will update the Figure legend to note that dot size conveys number of subjects which will influence IQR.***
